# Production of Monacolin K in *Monascus pilosus*: Comparison between Industrial Strains and Analysis of Its Gene Clusters

**DOI:** 10.3390/microorganisms9040747

**Published:** 2021-04-02

**Authors:** Weihua Dai, Yanchun Shao, Fusheng Chen

**Affiliations:** 1 Hubei International Scientific and Technological Cooperation Base of Traditional Fermented Foods, Huazhong Agricultural University, Wuhan 430070, China; dwh@webmail.hzau.edu.cn (W.D.); yanchunshao@mail.hzau.edu.cn (Y.S.); 2 College of Food Science and Technology, Huazhong Agricultural University, Wuhan 430070, China

**Keywords:** *Monascus pilosus*, genome, monacolin K, biosynthetic gene cluster

## Abstract

*Monascus pilosus* strains are widely applied to yield a cholesterol synthesis inhibitor monacolin K (MK), also called lovastatin (LOV). However, the mechanism of MK production by *M. pilosus* strains is still unclear. In this study, we firstly confirmed four *Monascus* strains, MS-1, YDJ-1, YDJ-2, and K104061, isolated from commercial MK products as *M. pilosus* and compared their abilities to produce MK in solid-state and liquid-state cultures. Then, we sequenced and analyzed their genomes and MK biosynthetic gene clusters (BGCs). The results revealed that the MK yields of MS-1, YDJ-1, YDJ-2, and K104061 in solid-state cultures at 14 days were 6.13, 2.03, 1.72, and 0.76 mg/g, respectively; the intracellular and extracellular MK contents of MS-1, YDJ-1, YDJ-2, and K104061 in liquid-state cultures at 14 days reached 0.9 and 1.8 mg/g, 0.38 and 0.43 mg/g, 0.30 and 0.42 mg/g, and 0.31 and 0.76 mg/g, respectively. The genome sizes of the four *M. pilosus* strains were about 26 Mb, containing about 7000–8000 coding genes and one MK gene cluster. The MK BGCs of MS-1, YDJ-2, and K104061 contained 11 genes, and the MK BGC of YDJ-1 contained 9 genes. According to the literature search, there are few comparisons of gene clusters and related genes responsible for the synthesis of LOV and MK. We also compared the LOV BGC in *A. terreus* with the MK BGCs in different species of *Monascus* spp., and the results revealed that although LOV and MK were the same substance, the genes responsible for the synthesis of MK were much less than those for LOV synthesis, and the gene functions were quite different. The current results laid a foundation to explore the mechanism of MK produced by *Monascus* spp. and compare the synthesis of LOV and MK.

## 1. Introduction

*Monascus* spp. are important filamentous fungi for foods and medicines, whose fermented rice product, Hongqu, also known as red yeast rice, has been used for nearly two thousand years in China and other Asian countries [1,2,3]. *Monascus* spp. can produce abundant secondary metabolites (SMs), such as *Monascus* pigments (MPs), monacolin K (MK), and γ-aminobutyric acid (GABA), and a few strains of *Monascus* spp. can also produce citrinin (CIT), a kidney mycotoxin [4,5,6,7,8,9], which leads to the safety issue of *Monascus* products. At present, the species commonly used in the production of Hongqu mainly belong to *M. pilosus*, *M. ruber*, and *M. purpureus* [10,11,12,13]. Research has revealed that the strains of *M. pilosus* can produce a large number of MK without CIT; thus, they are considered ideal producers for functional Hongqu [14,15]. Research has also shown that the different strains of *M. pilosus* can produce MK at various concentrations [16]. However, the mechanism of MK produced by *M. pilosus* is still unclear.

Fungal SMs mainly include polyketides (PKs), nonribosomal peptides (NRPs), and terpenes (TEs) [17,18], whose biosynthetic genes usually appear in the clusters [19]. PKs and NRPs are synthesized by polyketide synthase (PKS) and nonribosomal peptide synthetase (NRPS), respectively [20,21,22,23]. PKS can be divided into three types: Type I PKS, also called modular PKS, is mainly found in bacteria and fungi; Type II PKS, also known as aromatic PKS, mainly synthesizing aromatic compounds, exists only in bacteria; Type III PKS, also known as chalcone type PKS, mainly exists in plants, also in bacteria and few fungi [24,25]. PKs biosynthesized by Type I PKS may be the most abundant fungal SMs [19]. NRPS is a large-scale multimodule biocatalyst that utilizes complex reactions to produce peptide-based natural products, which are discovered in bacteria and fungi with huge chemical diversities and broad biological activities [26,27]. In addition, there also exist fungal PKS-NRPS hybrids that can produce a series of SMs with diverse structures with various biological functions [28]. By analyzing the key genes of PKS and NRPS in fungal genomes, it is possible to predict what SMs are mainly produced by fungi [19].

So far, eight genomes of *Monascus* strains (six in NCBI and two in the Joint Genome Institute (JGI)) have been released, of which five belong to *M. purpureus* and three belong to *M. ruber*. Up to now, no genome information of *M. pilosus* has been released, and few industrially applied strains of *Monascus* spp. are available on the genome [29,30,31,32].

In this study, first, based on the morphological classification methods, four strains of *Monascus* spp., MS-1, YDJ-1, YDJ-2, and K104061, isolated from different commercial Hongqu products [33,34], were identified as *M. pilosus*. Then, the yields of MK and CIT in solid-state and liquid-state cultures of the four strains were detected and compared. Furthermore, their genomes were sequenced, and their MK BGCs were predicted and analyzed based on their genomic information. 

## 2. Materials and Methods

### 2.1. Strains and Culture Conditions

Four strains of *Monascus* spp., MS-1, YDJ-1, YDJ-2, and K104061, isolated from Hongqu products were cultured on potato dextrose agar (PDA) media, respectively, and incubated at 28 °C for 7 days.

### 2.2. Preparation of the Spore Suspensions for Monascus spp. Strains

Sterile water was added to the PDA media slants of *Monascus* spp. strains, and spores were scraped off with an inoculation loop. Then, the spore fluids were poured into sterilized empty triangular flasks containing glass beads to disperse and were filtered with 2 to 3 layers of sterilized lens cleaning paper. After counting with a hemocytometer, the concentration of the spore suspension was adjusted to 10^6^/mL.

### 2.3. Classification and Identification of Monascus spp. Strains

#### 2.3.1. Colonial Morphologies of *Monascus* spp. Strains

A 0.5 μL volume of the spore suspensions of the four strains was, respectively, placed on the center of Petri dishes (Φ = 9 cm), including 4 media commonly used for *Monascus* spp.: malt extract agar (MA), Czapek yeast extract agar (CYA), potato dextrose agar (PDA), and 25% glycerol nitrate agar (G25N) [33,35]. Then, the Petri dishes were incubated at 28 ℃ for 7 days to observe the colonial morphologies of colony size and obverse and reverse colors of the colony and aerial hyphae. The colonial size was expressed as the average values of the colonial diameters in two vertical directions.

#### 2.3.2. Microscopic Morphologies of *Monascus* spp. Strains

A 200 μL volume of the spore suspensions of the four strains was spread on MA, CYA, PDA, and G25N media plates, respectively. The sterilized coverslips were inserted into the media at an angle of 45°. The plates were kept at 28 °C for 7 days, then the coverslips were placed under a microscope to observe the microscopic morphologies of mycelia, conidia, and cleistothecia.

#### 2.3.3. Molecular Identification of *Monascus* spp. Strains

Molecular identification of the strains was performed by alignment of their internal transcribed spacer (ITS) sequences. The four ITS sequences of *Monascus* strains were obtained by genome sequencing and blasted on the NCBI database, then sequences with higher homology were selected to construct the phylogenetic tree. The phylogenetic tree was generated in MEGA X using the Neighbor-Joining method [36,37]. Bootstrap values in the bootstrap test (1000 replicates) were shown above the branches [38]. The tree was drawn to scale, with branch lengths in the same units as those of the evolutionary distances used to infer the phylogenetic tree. The evolutionary distances were computed using the Kimura 2-parameter method [39] and were in the units of the number of base substitutions per site. 

### 2.4. Solid-State and Liquid-State Cultures of Monacus spp. Strains

#### 2.4.1. Culture Media

Seed culture medium: glucose, 50 g; peptone, 10 g; NH_4_H_2_PO_4_, 2.0 g; MgSO_4_·7H_2_O, 0.5 g; CaCl_2_, 0.1 g; potato juice to 1000 mL, pH 6.0.

Solid-state medium: rice flour, 30 g; soybean flour, 20 g; water content, 35%; acetic acid, 0.6% (*v*/*w*); MgSO_4_·7H_2_O, 0.004 mol/kg; after mixing, sterilizing and cooling.

Liquid-state medium: potato juice was taken as the basic medium, soybean flour, 38.75 g/L; sucrose, 30 g/L; MgSO_4_·7H_2_O, 0.00105 mol/L; pH 5.5; sterilizing and cooling [33].

#### 2.4.2. Preparation of Seed Liquid

The spore suspensions prepared in Section 2.2 were inoculated into the seed culture medium with 10% (*v/v*) inoculum and cultured on a shaker at 150 rpm and 30 °C for 30 h [33].

#### 2.4.3. Solid-State and Liquid-State Cultures

Seed liquid (10% (*v/v*)) was inoculated in the solid-state media, cultured at 30 °C for 60 h, then transferred to 24 °C to continue to be cultured for 14 days [34]. The samples were taken on the 4th, 8th, 12th, and 14th days then dried at 45 °C and crushed.

Seed liquid (10% (*v/v*)) was inoculated in the liquid-state media, cultured at 30 °C and 110 rpm for 3 days, then transferred to 25 °C to continue to culture for 14 days. The samples were taken on the 4th, 8th, 12th, and 14th days [33].

### 2.5. MK and CIT Analysis

#### 2.5.1. MK and CIT Extraction of Solid-State Samples

MK extraction: 0.1 g of dried solid-state samples were dried to a constant weight at 40 °C, suspended in 10 mL of 75% (*v*/*v*) ethanol solution, and subjected to ultrasonication treatment (KQ-250B, Kunshan, China) for 60 min then centrifuged at 8000 rpm for 15 min. The supernatant was collected and filtered through a 0.22 μm microfiltration membrane.

CIT extraction: 0.3 g of dried solid-state samples were suspended in 3 mL of 80% (*v*/*v*) methanol solution and subjected to ultrasonication treatment for 40 min then centrifuged at 8000 rpm for 15 min, and the supernatant was gathered. Another 3 mL of 80% methanol was added to the precipitate. Both supernatants were combined, diluted to 10 mL, and filtered through a 0.22 μm microfiltration membrane after ultrasonic extraction for 20 min and centrifugation. 

#### 2.5.2. MK and CIT Extraction of Liquid-State Samples

Intracellular MK/CIT extraction: 0.1 g/0.3 g of freeze-dried mycelia were taken, and the extraction steps are the same as the MK/CIT extraction of the solid-state samples in Section 2.5.1.

Extracellular MK extraction: after the mycelia were filtered, absolute ethanol was added at the ratio of 1:3 (fermentation broth/absolute ethanol). After standing still for 30 min, the mixture was centrifuged at 10,000 rpm for 10 min. The supernatant was collected and filtered through a 0.22 μm microfiltration membrane.

Extracellular CIT extraction: the mycelia were filtered to obtain the clarified fermentation broth, which was filtered through a 0.22 μm microfiltration membrane for further analysis. 

#### 2.5.3. MK and CIT Detection

MK and CIT contents were detected by high-performance liquid chromatography (HPLC, Shimadzu LC-20AT, Kyoto, Japan), equipped with a C_18_ column (inertsil ODS-3 4.6 × 250 mm) by means of a diode array detector. A 20 μL volume of sample extract solution was injected into HPLC to detect MK and CIT. Both acid and lactone forms of MK were calculated as the MK yield. The detailed HPLC parameters were as follows. 

For MK detection, solvent A: acetonitrile; solvent B: 0.05% phosphoric acid in water; 60% A and 40% B for 30 min; detection wavelength: 238 nm; column temperature: 40 °C, flow rate: 1.0 mL/min.

For CIT detection, solvent A: acetonitrile; solvent B: 0.1% phosphoric acid in water; 70% A and 30% B for 20 min; detection wavelength: 331 nm; column temperature: 30 °C, flow rate: 0.8 mL/min.

#### 2.5.4. Statistical Analyses

Statistical analyses were performed with SPSS (version 16.0) to calculate the means, standard errors, and standard deviations. The statistical significance was calculated by one-way analysis of variance (ANOVA), with significance levels set at *p* = 0.05.

### 2.6. Genomic DNA Extraction

Four *Monascus* spp. strains, MS-1, YDJ-1, YDJ-2, and K104061, were, respectively, cultured on PDA plates at 28 °C for 4 days, then mycelia were collected and stored at −80 °C for genomic DNA extraction using the CTAB method [40]. 

### 2.7. DNA Sequencing and Assembly

DNA samples prepared in Section 2.6 were randomly broken into fragments of the length required to construct DNA libraries. NEBNext^®^ Ultra™ DNA Library Prep Kit for Illumina and SMRT bell TM Template kit 1.0 were used to construct the Illumina library and 20K SMRT Bell library, respectively. Illumina NovaSeq PE150 and PacBio Sequel platforms were applied for the whole-genome sequencing, then the raw data were valued by FastQC [41], and SMRT Link v5.1.0 software [42,43] was utilized to assemble genomes.

### 2.8. Annotation and Analysis of Monascus spp. Genomes

Based on the sequence information of the four strains in this research and other genomes of *Monascus* spp., which have been released in NCBI and JGI, prediction of the coding genes was performed with Augustus [44]. The SM BGCs were predicted by AntiSMASH 5 for fungi [45]. The Pfam database (http://pfam.xfam.org/, accessed on 3 December 2019) and the conserved domain database (CDD, https://www.ncbi.nlm.nih.gov/cdd, accessed on 3 September 2020) were used to predict and analyze the conserved domains to re-predict the gene functions in the LOV and MK BGCs. The Geneious software [46] was used to analyze the sequence similarity of the genes in the LOV and MK BGCs by the Geneious alignment method, and the parameters were set as default.

### 2.9. Data Deposition

The four genomes of *M. pilosus,* MS-1, YDJ-1, YDJ-2, and K104061, could be obtained from NCBI (BioProject Accession Number: PRJNA718072).

## 3. Results

### 3.1. Classification and Identification

All four strains of *Monascus* spp., MS-1, YDJ-1, YDJ-2, and K104061, used in the current study were from the production plants of *Monascus* products [33,34], so their taxonomic status was reidentified based on the morphological and molecular identification methods.

#### 3.1.1. Morphological Identification of *Monascus* spp.

**Figure 1:**
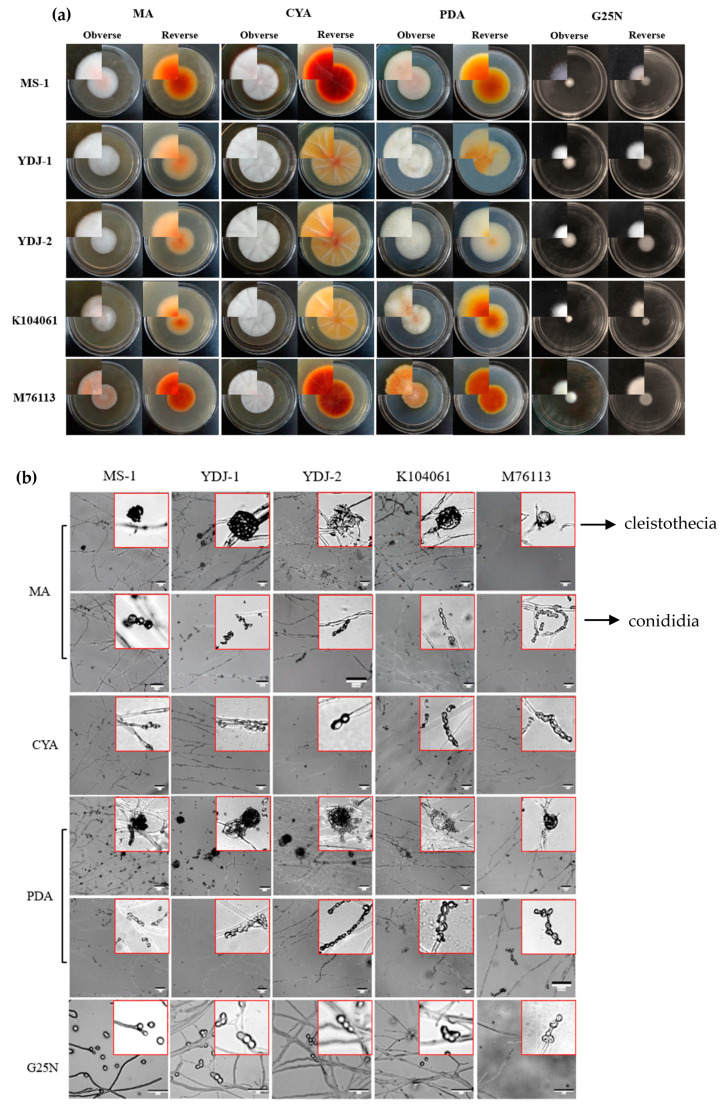
Colonial and microscopic morphologies of *Monascus* spp. strains on different media at 28 °C for 7 days. (**a**) Colonial morphologies; (**b**) microscopic morphologies. Scale bars: 50 nm; the images in the red frame were magnified images of cleistothecia or conidia.

As shown in Figure 1 and Table 1, on the seventh day, on MA media, the reverse surfaces of colonies were yellow at the margins and deep orange at the centers; on CYA media, colonies were irregular in shape; on PDA media, the edges of colonies were light yellow to golden yellow; on G25N media, colonies were floccose, mycelia were white, and the reverse was uncolored. The microscopic morphologies of the four strains showed that there were cleistothecia and conidia on MA and PDA media, while only conidia could be observed on CYA and G25N media. The morphological characteristics of the strains were similar to those described in the literature of *M. pilosus* [35,47].

#### 3.1.2. Molecular Identification of *Monascus* spp. Strains

The phylogenetic analysis in this study used ITS sequences (the four ITS sequences of *Monascus* strains were obtained by genome sequencing, and the other ITS sequences were from NCBI). The evolutionary history was inferred using the Neighbor-Joining method. Bootstrap values were above branches, and only those above 60% were indicated. The strains of *Penicillium griseum* and *Aspergillus fischeri* which were farther from the tested strains were used as the outgroup.

**Figure 2:**
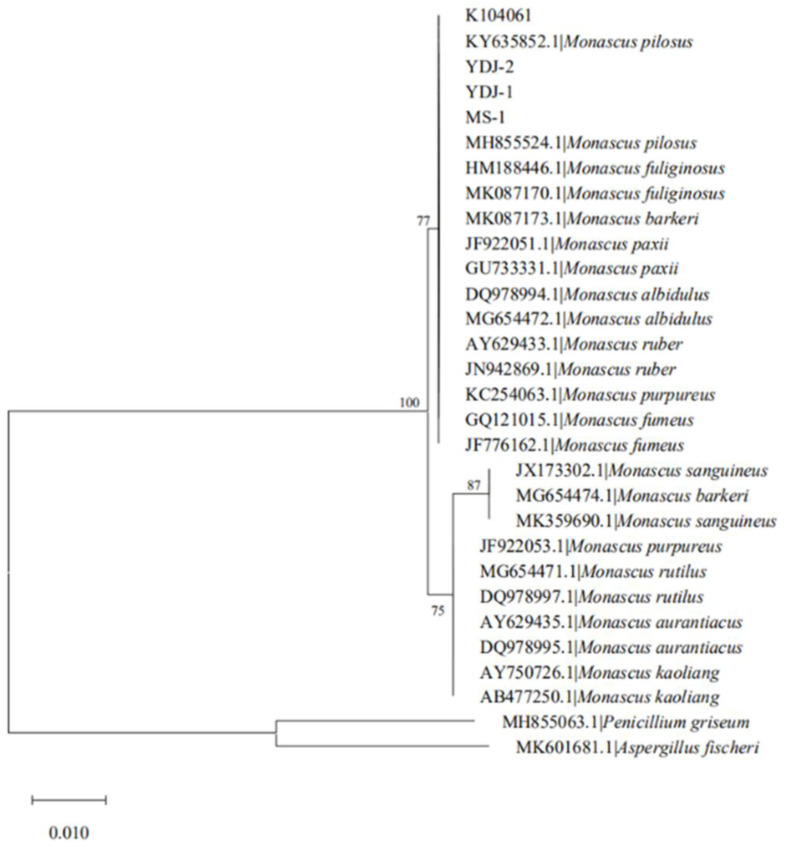
Phylogenetic tree from analysis with internal transcribed spacer (ITS) sequences of *Monascus* spp.

As shown in Figure 2, the four *Monascus* strains were clustered with *M. pilosus*, *M. fuliginosus*, *M. barkeri*, *M. paxii*, *M. albidulus*, *M. ruber*, *M. purpureus*, and *M. fumeus* and very close to *M. pilosus*, indicating that they were *M. pilosus*.

Combined with the results of morphological and molecular identification, the *Monascus* strains, MS-1, YDJ-1, YDJ-2, and K10406, isolated from factories and used in this study were identified as *M. pilosus*.

### 3.2. MK and CIT Production in Solid-State and Liquid-State Cultures

According to the formula of solid and liquid culture media, stage-variable temperature culture was used and samples were taken on the 4th, 8th, 12th, and 14th days of the culture process.

**Figure 3:**
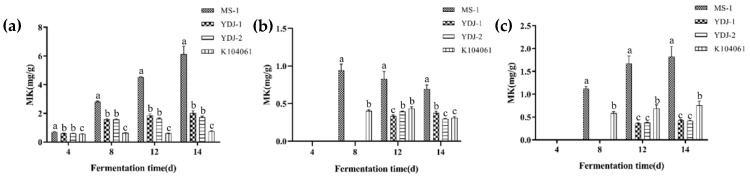
Monacolin K (MK) contents in solid-state and liquid-state cultures. (**a**) MK content of the four strains during solid culture; (**b**) intracellular MK content of the four strains during liquid culture; (**c**) extracellular MK content of the four strains during liquid culture.

As shown in Figure 3, MS-1 could produce the highest concentration of MK in both solid-state and liquid-state cultures. In solid-state cultures at 14 days, the MK yields of, MS-1, YDJ-1, YDJ-2, and K104061 reached 6.13, 2.03, 1.72, and 0.76 mg/g, respectively. In liquid-state cultures at 14 days, the intracellular and extracellular MK contents of MS-1, YDJ-1, YDJ-2, and K104061 were 0.9 and 1.8 mg/g, 0.38 and 0.43 mg/g, 0.30 and 0.42 mg/g, and 0.31 and 0.76 mg/g, respectively. CIT was detected neither in solid-state nor in liquid-state cultures for all tested *Monascus* strains (data not shown).

### 3.3. Genome Sequencing and Prediction of SM Gene Clusters

The four *Monascus* strains were sequenced and analyzed. The results (Table 2) showed that the genome sizes were roughly 26 Mb, the G+C mole percentages were approximately 49%, and the coding genes varied from 7634 to 7771, respectively.

AntiSMASH 5 was used to predict the SMs gene clusters in the four genomes, and the results (Table S1) showed that five types of SMs were predicted. Furthermore, the MK BGCs appeared in their genomes but no CIT BGCs. 

### 3.4. Comparison of MK BGCs

#### 3.4.1. Function Re-Prediction of the Genes in the LOV and MK BGCs Reported Previously

In 1999, Kennedy [48] reported a BGC related to LOV BGC in the genome of *Aspergillus terreus* ATCC 20542, which contained total 18 genes including 7 unknown functional ones at that time (Table 3), and in 2013, Xu et al. renamed one unknown gene, *ORF5* as *lovG* [49]. In 2008, Chen and collaborators [50] reported a BGC related to monocolin K (MK BGC) in *M. pilosus* BCRC38072, which only contained 9 genes (Table 3). In this research, we re-predicted functions of genes in LOV and MK BGCs by Pfam and CDD analysis, the results showed that in LOV BGC, all of *ORF2*, *ORF10,* and *ORF16* were re-predicted as transporters, *ORF14*, *ORF15,* and *ORF18* were re-predicted as mitochondrial carrier protein, dehydratase and glycosyl hydrolase, respectively, while *ORF12* was still unknown, and *lovG* and *mkD* were re-predicted as α/β hydrolase (Table 3).

#### 3.4.2. Comparison of MK BGCs

**Figure 4:**
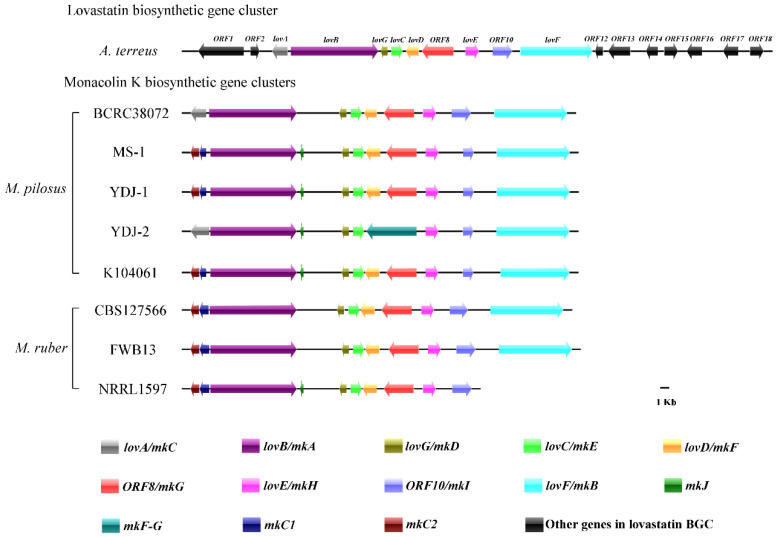
Genes involved in the biosynthesis of LOV or MK. The LOV and MK BGCs were obtained from the GenBank database (Accession Numbers: AH007774.2 and AF151722 for LOV BGC; DQ176595.1 for MK BGC).

Based on the LOV and MK BGCs in Table 3, we compared twelve genomes of *Monascus* spp. including eight published genomes, *M. purpureus* YY-1, YJX-8, GB-01, HQ1, NRRL1596, and *M. ruber* FWB13, CBS127566, NRRL 1597 [29,30,31,32], and four genomes of *M. pilosus* sequenced in this study, and found that all genomes of four *M. pilosus* strains and three *M. ruber* strains contained the MK BGCs while there was no MK BGC in those of five *M. purpureus* strains. Total 9 or 10 genes named *mkA-mkI* were highly conserved in the LOV and MK BGCs (Table 4, Figure 4). However, there were some unique genes in MK BGCs of *Monascus* spp. For example, in YDJ-2, Both of *mkF* and *mkG* were combined into be one gene *mkF-G*, and *mkC* in *M. pilosus* BCRC38072 [50] was predicted to be one gene *mkC* in YDJ-1 or to be two independent genes *mkC1* and *mkC2* in other MK BGCs. In addition, there was an extra gene *mkJ* in the MK BGCs of MS-1, YDJ-1, YDJ-2, K104061, and NRRL1597, which neither existed in the LOV BGC of *A. terreus* ATCC 20542 [48] nor in the MK BGCs of FWB13, CBS127566, and BCRC38072. Based on KOG annotation result obtained from JGI, MKJ was one animal-type fatty acid synthase and related protein. It was worth noting that although *lovF/mkB* did not exist in the MK BGCs of NRRL1597, it was located elsewhere in its genome, and this situation also occurred in *mkJ* in the FWB13 and CBS127566 genomes (data not shown).

#### 3.4.3. Multiple sequence alignment of LOV and MK BGCs

**Figure 5:**
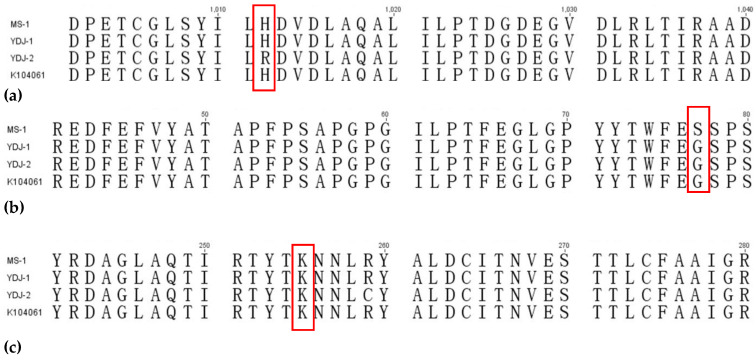
Amino acid mutations in the four MK BGCs from *M. pilosus*, MS-1, YDJ-1, YDJ-2, and K104061. The Geneious software was used to perform multiple sequence alignment. (**a**) The histidine at position 1012 of MKB was mutated to arginine in YDJ-2; (**b**) the glycine at position 77 of MKD was mutated to serine in MS-1; (**c**) the arginine at position 259 of MKE was mutated to cysteine in YDJ-2. The content in the red frames represented the type of mutant amino acid and its positions in the corresponding proteins.

A multiple sequence alignment of each gene in LOV and MK BGCs (Figure 4) revealed that genes in MK BGCs were quite different from those in LOV BGC, and the genes in MK BGCs from the same species of *Monascus* spp. showed higher homology (Figures S1–S10). Among MK BGCs of the strains of *M. pilosus*, the 1012th histidine of MKB in YDJ-2 was mutated to arginine (Figure 5a), and the 77th glycine of MKD in MS-1 was mutated to serine (Figure 5b). The arginine at position 259 of MKE was mutated to cysteine in YDJ-2 (Figure 5c).

Further, we analyzed if these amino acid mutations occurred on the active or binding sites of MKB in YDJ-2, MKD in MS-1, and MKE in YDJ-2 and affected their functions. We found that the amino acid mutations in MKB, MKD, and MKE did not occur in the active or binding sites (Figures S11–S13, Table S2) and did not affect their functions. 

## 4. Discussion

In 1979, Endo identified a substance from the fermentation broth of *M. ruber* and named it MK that could inhibit cholesterol synthesis [51]. In 1980, Albert [52] discovered a similar substance from *A. terreus* and named it mevinolin. Subsequent research has proven that mevinolin and MK are the same substance, and now, both of them are often referred to collectively as lovastatin (LOV) [53]. Although MK and LOV are the same substance, there were also some differences among the MK BGCs of *Monascus* spp. and LOV BGC. In addition, different species of *Monascus* spp. contained different MK BGCs, and there were also differences among genes related to MK synthesis in *Monascus* spp. In this research, the SM BGC prediction results of *Monascus* spp. showed that the strains of *M. ruber* and *M. pilosus* contained MK BGCs. There were 10 genes in each MK BGC of *M. ruber* FWB13, CBS127566, and NRRL 1597. Among *M. pilosus* strains, there were 11 genes in each MK BGC of MS-1, YDJ-1, and K104061 and 9 genes in the MK BGC of YDJ-2 (Table 4, Figure 4), while 18 genes were reported responsible for the LOV biosynthesis [48], in which there were 9 genes that may be essential and conserved genes for the biosynthesis of MK. In the MK BGC of *M. ruber* NRRL 1597, *mkB* was absent, but there was an extra gene, *mkJ*, which was related to the synthesis of animal-type fatty acid; *mkC* in *M. pilosus* BCRC38072 and YDJ-1 were predicted to be two independent genes *mkC1* and *mkC2* in other strains of *Monascus* spp., whose functions were the same as *mkC*; in YDJ-2, *mkF* and *mkG* were combined into one gene, *mkF-G*, with the functions of both of *mkF* and *mkG*.

The MK BGCs of the four strains of *M. pilosus* studied in this research contained the key genes related to the MK synthesis [50]; thus, theoretically, all four strains had the ability to produce MK. According to the results of solid-state and liquid-state cultures, all strains of MS-1, YDJ-1, YDJ-2, and K104061 could indeed produce MK at various concentrations, and MS-1 had the strongest ability to yield MK (Figure 3). The results of multiple sequence alignment revealed that there were amino acid mutations in MKB of YDJ-2, MKD of MS-1, and MKE of YDJ-2 (Figure 5), but these mutations did not occur at the active or binding sites of these proteins (Figures S11–S13, Table S2). We further analyzed the transcription of these genes in the four MK BGCs, and the results showed that each gene was expressed to varying degrees (data not shown), which could not figure out the reason for the difference to yield MK of the four strains, yet. However, the effect of a mutation also depends on the position of the amino acid in the 3D structure [54]. Later, the 3D structure of the mutant proteins can be simulated and compared by related experiments to further explore the effect of the mutations on the function of these proteins. In addition, transcriptomic analysis or other methods should be used to explore the differences in MK production of the four strains. 

In conclusion, all *Monascus* strains, MS-1, YDJ-1, YDJ-2, and K104061, were identified as *M. pilosus* and had the ability to produce MK. The MK BGCs identified in the four strains involved 9–11 genes, in which 9 essential genes responsible for the MK biosynthesis were highly conserved in *M. pilosus*. Genes responsible for the synthesis of MK were much less than those of LOV, whose functions were also not the same. The results of this study may provide a theoretical basis to explore the mechanism of MK produced by *Monascus* spp. and compare the synthesis of LOV and MK.

## Figures and Tables

**Table 1:** Morphologic characteristics of *Monascus* spp. on different media for 7 days.

	MA	CYA	PDA	G25N
**Colonial Morphologies**	The colonial diameters reached 40–48 mm. Their obverse and reverse surfaces were white and light orange to orange-red, respectively. Their mycelia were compact and dense.	The colonial diameters reached 50–65 mm. Their shapes were irregular. Their obverse and reverse surfaces were white and red to dark red with radial folds. Their mycelia were sparse and short.	The colonial diameters reached 40–55 mm. Their reverse surfaces were light orange or orange-red and the edges were light yellow to golden yellow. Their middle parts were raised, and their mycelia were dense and fluffy.	The colonial diameters reached 11–25 mm. Their obverse and reverse surfaces were white and colorless.
**Microscopic Morphologies**	Cleistothecia arose singly at the tips of stalk-like hyphae and walls were hyaline or pale brown. Conidia were hyaline and borne laterally on pedicels and terminally on hyphae, arising singly or occasionally in short chains, obpyriform to globose.	No cleistothecium; conidia were transparent or brown and obpyriform to globose.	Cleistothecia were globose and arose singly from distinct stalk-like hyphae. Conidia were spherical or upside-down pear-shaped with colorless or brown colors.	No cleistothecium; conidia were spherical, transparent, and colorless.

**Table 2:** General features of genomic information of four *M. pilosus* strains.

Genome Features	MS-1	YDJ-1	YDJ-2	K104061
Genome length (Mb)	26.21	26.15	26.16	26.14
GC content (%)	48.89	48.90	48.89	48.87
Gene amount (#)	7771	7687	7718	7634
Gene total length (Mb)	13.26	13.04	13.13	13.09
Gene average length (bp)	1707	1696	1701	1715
Gene length/Genome (%)	50.60	49.86	50.18	50.08

**Table 3:** Re-prediction of functions of genes in lovastatin (LOV) and MK biosynthetic gene clusters (BGCs).

lov/mk^*^	Function (Previous Study)	Function (Re-Predicted in This Study)
*lovA/mkC*	Cytochrome P450 monooxygenase	Cytochrome P450 monooxygenase
*lovB/mkA*	LOV nonaketide synthase	LOV nonaketide synthase
*lovC/mkE*	Enoyl reductase/Dehydrogenase	Enoyl reductase/Dehydrogenase
*lovD/mkF*	Transesterase	Transesterase
*lovE/mkH*	Regulatory protein	Regulatory protein
*lovF/mkB*	LOV diketide synthase	LOV diketide synthase
***lovG/mkD***	**Thioesterase/Oxidoreductase**	**α/β hydrolase**
*ORF1/-*	Esterase	Esterase
***ORF2/-***	**Unknown**	**Transporters**
*ORF8/mkG*	HMG-CoA reductase	HMG-CoA reductase
***ORF10/mkI***	**Unknown**/Efflux pump	**Transporters**
***ORF12/-***	**Unknown**	**Unknown**
*ORF13/-*	Regulatory protein	Regulatory protein
***ORF14/-***	**Unknown**	**Mitochondrial carrier protein**
***ORF15/-***	**Unknown**	**Dehydratase**
***ORF16/-***	**Unknown**	**Transporters**
*ORF17/-*	Cytochrome P450 monooxygenase	Cytochrome P450 monooxygenase
***ORF18/-***	**Unknown**	**Glycosyl hydrolase**

* Genes whose functions were different from the previous functions in this research are shown in bold. “-”: The relative genes could not be found in MK BGC.

**Table 4:** Homologous comparison of the genes in LOV and MK BGCs (%).

*lov/mk* Genes	*M. pilosus*				*M. ruber*		
	MS-1	YDJ-1	YDJ-2	K104061	FWB13	CBS127566	NRRL1597
*lovB/mkA*	76.75/99.93	76.75/99.93	76.75/99.93	76.75/99.93	76.75/99.93	76.65/99.80	76.81/100
*lovF/mkB*	72.23/99.96	72.57/99.96	72.61/100	72.23/99.96	71.48/98.44	71.52/98.44	-
*lovA/mkC*	67.14/88.73 92.11/100	*-/-* 84.57/97.91	67.14/88.73 92.11/100	67.14/88.73 92.11/100	67.14/88.73 92.11/100	67.14/88.73 92.11/100	67.61/88.73 *mkC1* 92.11/100 *mkC2*
*lovG/mkD*	67.16/99.62	66.79/100	66.79/100	66.79/100	64.18/100	64.18/96.58	64.18/100
*lovC/mkE*	78.01/93.02	78.01/93.02	77.75/92.76	78.01/93.02	78.59/94.17	78.59/94.17	78.59/94.17
*lovD/mkF*	87.41/97.28	88.17/100	87.93/96.25	87.41/97.28	86.90/97.02	86.90/97.02	86.90/97.02
*ORF8/mkG*	71.98/100	71.72/99.62	71.98/100	71.98/100	71.98/100	71.98/100	71.98/100
*lovE/mkH*	55.65/100	55.65/100	55.65/100	55.65/100	55.65/100	55.86/99.77	55.44/99.32
*ORF10/mkI*	79.26/100	79.26/100	79.26/100	79.26/100	78.31/97.24	79.75/96.82	78.31/97.24

The numbers with “%” are the similarity percentages between *mkA*-*mkI* from *Monascus* spp. investigated in this study and the corresponding genes in the LOV/MK BGCs reported previously [48,50].

## Data Availability

The raw data supporting the conclusions of this manuscript will be made available by the authors, without undue reservation, to any qualified researcher.

## Supplementary Materials

The following are available at https://www.mdpi.com/article/10.3390/microorganisms9040747/s1, Table S1: Prediction of secondary metabolite gene clusters of four *M. pilosus* strains, Table S2: S2-1 Active sites in MKB of the MK biosynthetic gene cluster in MS-1, S2-2: Binding sites in MKB of the MK biosynthetic gene cluster in MS-1, S2-3: Binding sites in MKE of the MK biosynthetic gene cluster in MS-1. Figure S1: Multiple sequence alignment of gene *mkA*, Figure S2: Multiple sequence alignment of gene *mkB*, Figure S3: Multiple sequence alignment of gene *mkC1*, Figure S4: Multiple sequence alignment of gene *mkC2*, Figure S5: Multiple sequence alignment of gene *mkD*, Figure S6: Multiple sequence alignment of gene *mkE*, Figure S7: Multiple sequence alignment of gene *mkF*, Figure S8: Multiple sequence alignment of gene *mkG*, Figure S9: Multiple sequence alignment of gene *mkH*, Figure S10: Multiple sequence alignment of gene *mkI*. Figure S11: Prediction of active and binding sites in MKB, Figure S12: Prediction of active and binding sites in MKD, Figure S13: Prediction of active and binding sites in MKE.
