# Supplementary material for: Production of Monacolin K in Monascus pilosus: Comparison between Industrial Strains and Analysis of Its Gene Clusters
Source: Microorganisms. 2021 Apr 2;9(4):747. doi: 10.3390/microorganisms9040747 (PMC8065618; doi:10.3390/microorganisms9040747)
Supplement: Supplementary file 1 [file microorganisms-09-00747-s001.zip › microorganisms-1152183-supplementary-published (final)/Table S1.docx]

**Table S1 Prediction of secondary metabolite gene clusters of four *M. pilosus* strains***

| Sample ID | Cluster type | Cluster number | Function of *t1pks* gene clusters | | | | |
| --- | --- | --- | --- | --- | --- | --- | --- |
|  |  |  | **MK** | **MPs** | **CIT** | **TAN-1612** | **T4HN** |
| Ms-1 | *t1pks* | 7 | √ | √ |  |  | √ |
|  | *nrps* | 7 |  |  |  |  |  |
|  | *t1pks-nrps* | 4 |  |  |  |  |  |
|  | *terpene* | 2 |  |  |  |  |  |
|  | *beta-lactone* | 2 |  |  |  |  |  |
|  | *other* | 5 |  |  |  |  |  |
| YDJ-1 | *t1pks* | 5 | √ | √ |  |  |  |
|  | *nrps* | 7 |  |  |  |  |  |
|  | *t1pks-nrps* | 4 |  |  |  |  |  |
|  | *terpene* | 2 |  |  |  |  |  |
|  | *beta-lactone* | 2 |  |  |  |  |  |
|  | *other* | 5 |  |  |  |  |  |
| YDJ-2 | *t1pks* | 6 | √ | √ |  | √ |  |
|  | *nrps* | 7 |  |  |  |  |  |
|  | *t1pks-nrps* | 3 |  |  |  |  |  |
|  | *terpene* | 2 |  |  |  |  |  |
|  | *beta-lactone* | 2 |  |  |  |  |  |
|  | *other* | 5 |  |  |  |  |  |
| K104061 | *t1pks* | 6 | √ | √ |  |  |  |
|  | *nrps* | 7 |  |  |  |  |  |
|  | *t1pks-nrps* | 4 |  |  |  |  |  |
|  | *terpene* | 2 |  |  |  |  |  |
|  | *beta-lactone* | 2 |  |  |  |  |  |
|  | *other* | 5 |  |  |  |  |  |

*MK: monacolin K; MPs: *Monascus* pigments; CIT: citrinin; T4HN: 1,3,6,8-tetrahydroxynaphthalene.
