# Supplementary material for: Production of Monacolin K in Monascus pilosus: Comparison between Industrial Strains and Analysis of Its Gene Clusters
Source: Microorganisms. 2021 Apr 2;9(4):747. doi: 10.3390/microorganisms9040747 (PMC8065618; doi:10.3390/microorganisms9040747)
Supplement: Supplementary file 1 [file microorganisms-09-00747-s001.zip › microorganisms-1152183-supplementary-published (final)/Table S2.docx]

**Table S2-1.** Active sites in MKB of the MK biosynthetic gene cluster in MS-1

| Amino acid position | Type |
| --- | --- |
| 87 | Cysteine |
| 222、257 | Histidine |
| 2181 | Lysine |
| 2203 | Serine |
| 2216 | Tyrosine |
| 2220 | Glycine |

**Table S2-2.** Binding sites in MKB of the MK biosynthetic gene cluster in MS-1

| Amino acid position | Type |
| --- | --- |
| 1345、1400、1964、1989、1991 | Isoleucine |
| 1346、1347、1348、1350、1351、1871、1874、1875、1896、2042 | Glycine |
| 1349、1850 | Threonine |
| 1373、1399、1990 | Aspartic acid |
| 1374、1876 | Valine |
| 1398 | Leucine |
| 1420 | Cysteine |
| 1766 | Phenylalanine |
| 1873、1895 | Alanine |
| 1900 | Lysine |
| 1918、1942 | Serine |
| 1919 | Arginine |
| 2039 | Methionine |
| 2040 | Glutamine |
| 2044 | Histidine |

**Table S2-3.** Binding sites in MKE of the MK biosynthetic gene cluster in MS-1

| Amino acid position | Type |
| --- | --- |
| 49 | Proline |
| 50、173、223、378 | Serine |
| 138、174、176、306 | Threonine |
| 175 | Alanine |
| 222、264 | Cysteine |
| 225 | Histidine |
| 226、289 | Asparagine |
| 241 | Tyrosine |
| 265 | Isoleucine |
| 288 | Leucine |
| 308、379 | Glycine |
