# Supplementary figures and images for: Production of Monacolin K in Monascus pilosus: Comparison between Industrial Strains and Analysis of Its Gene Clusters
Source: Microorganisms. 2021 Apr 2;9(4):747. doi: 10.3390/microorganisms9040747 (PMC8065618; doi:10.3390/microorganisms9040747)

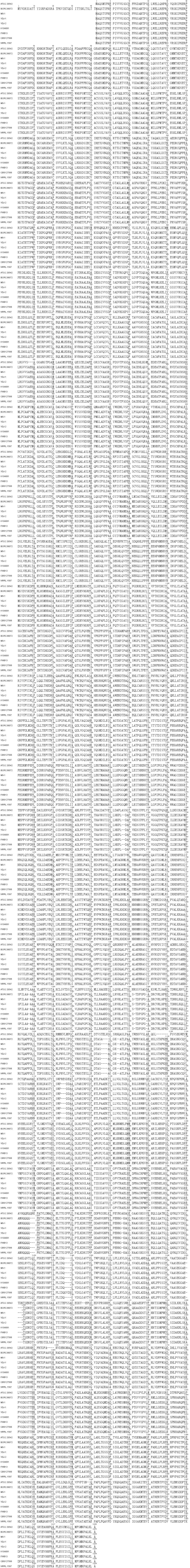

Supplement: Supplementary file 1 [file microorganisms-09-00747-s001.zip › microorganisms-1152183-supplementary-published (final)/Figure S1.jpg]

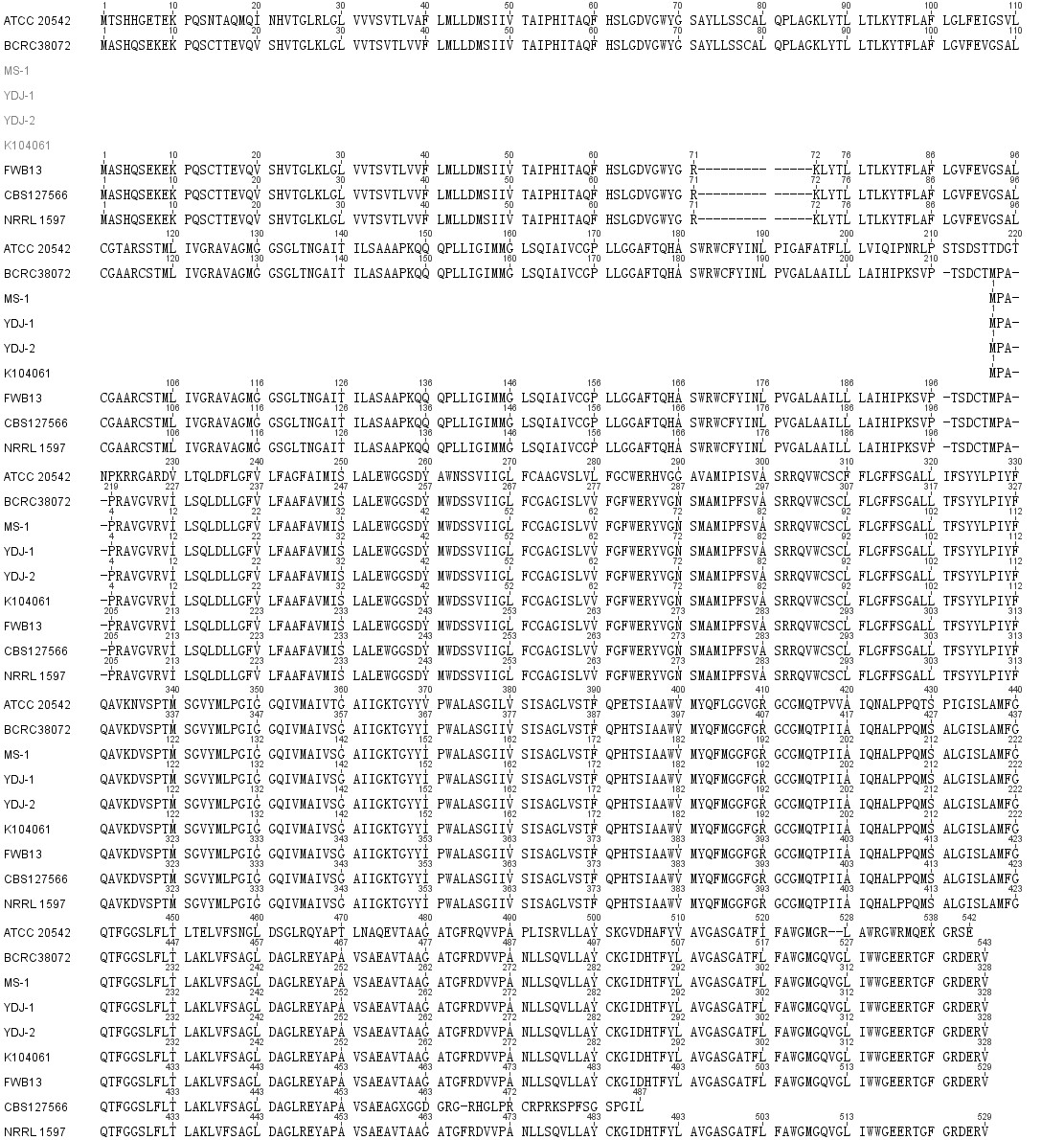

Supplement: Supplementary file 1 [file microorganisms-09-00747-s001.zip › microorganisms-1152183-supplementary-published (final)/Figure S10.jpg]

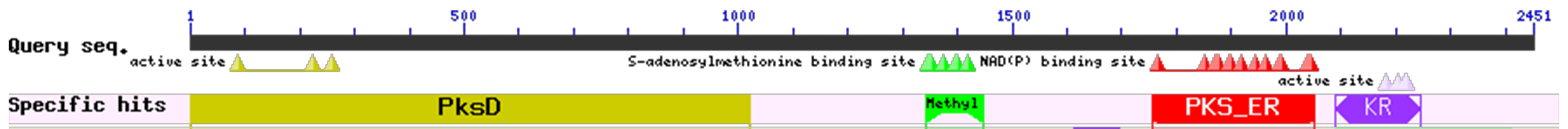

Supplement: Supplementary file 1 [file microorganisms-09-00747-s001.zip › microorganisms-1152183-supplementary-published (final)/Figure S11.tif]

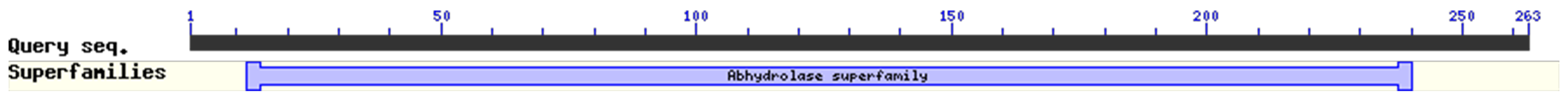

Supplement: Supplementary file 1 [file microorganisms-09-00747-s001.zip › microorganisms-1152183-supplementary-published (final)/Figure S12.tif]

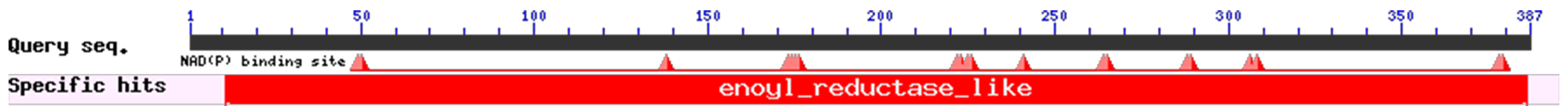

Supplement: Supplementary file 1 [file microorganisms-09-00747-s001.zip › microorganisms-1152183-supplementary-published (final)/Figure S13.tif]

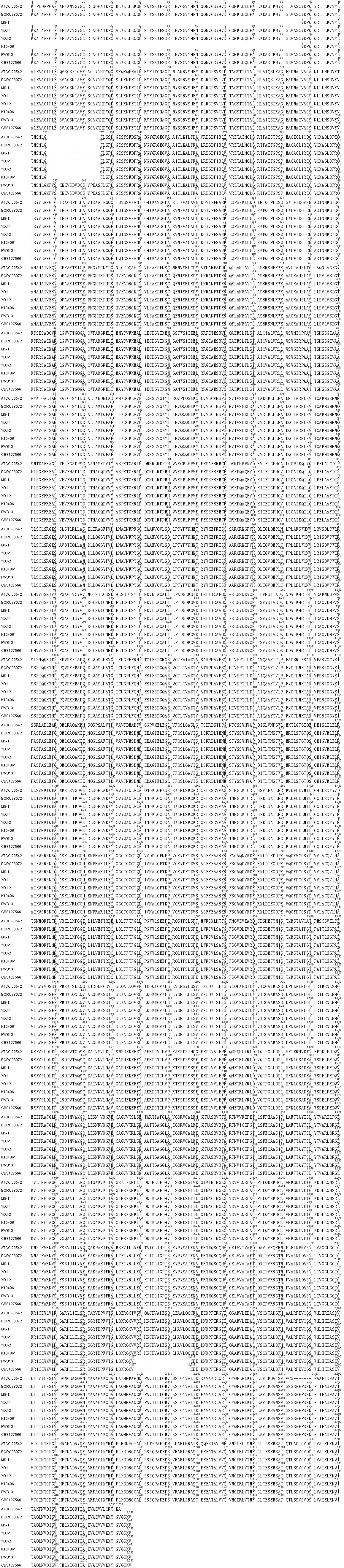

Supplement: Supplementary file 1 [file microorganisms-09-00747-s001.zip › microorganisms-1152183-supplementary-published (final)/Figure S2.jpg]

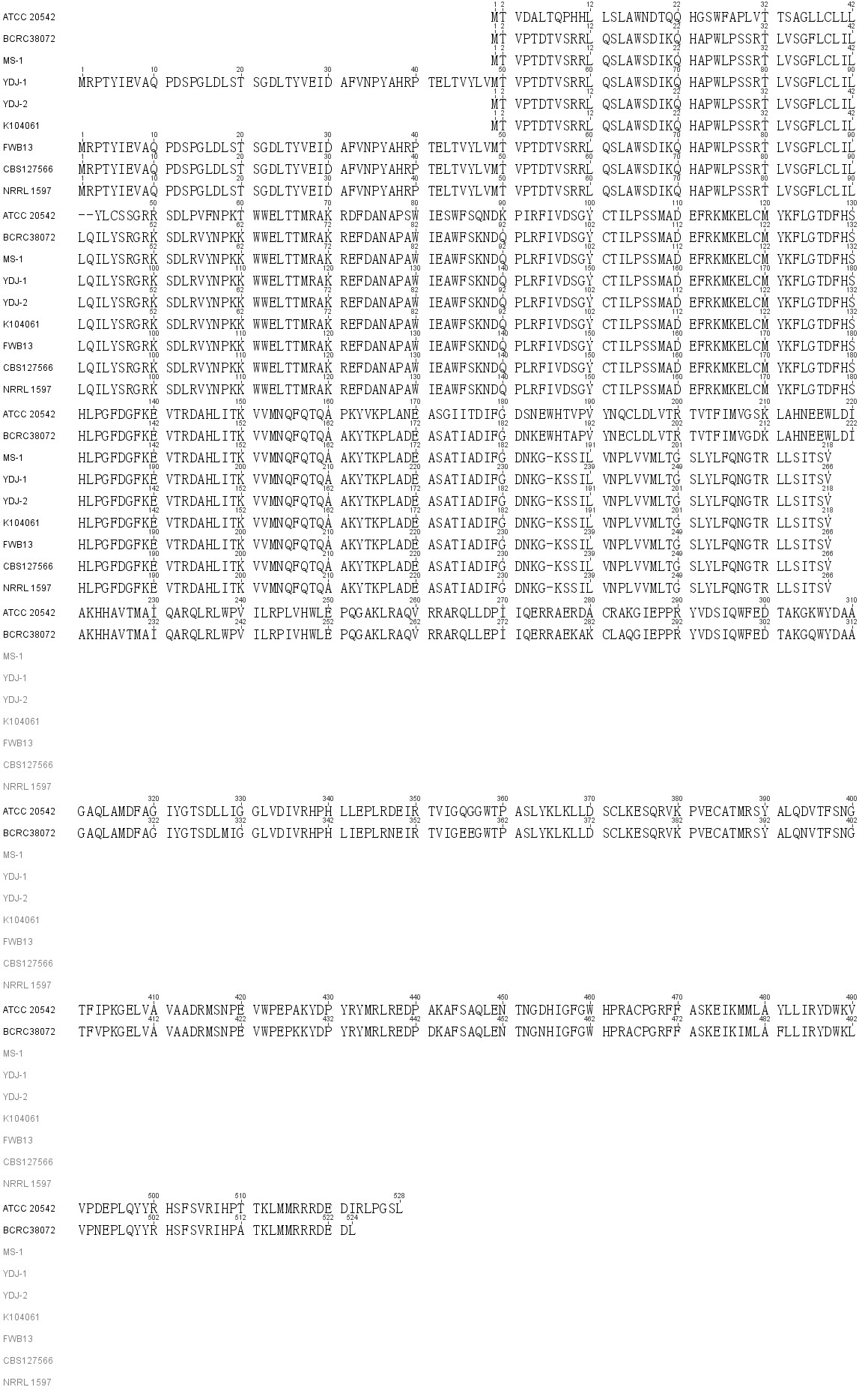

Supplement: Supplementary file 1 [file microorganisms-09-00747-s001.zip › microorganisms-1152183-supplementary-published (final)/Figure S3.jpg]

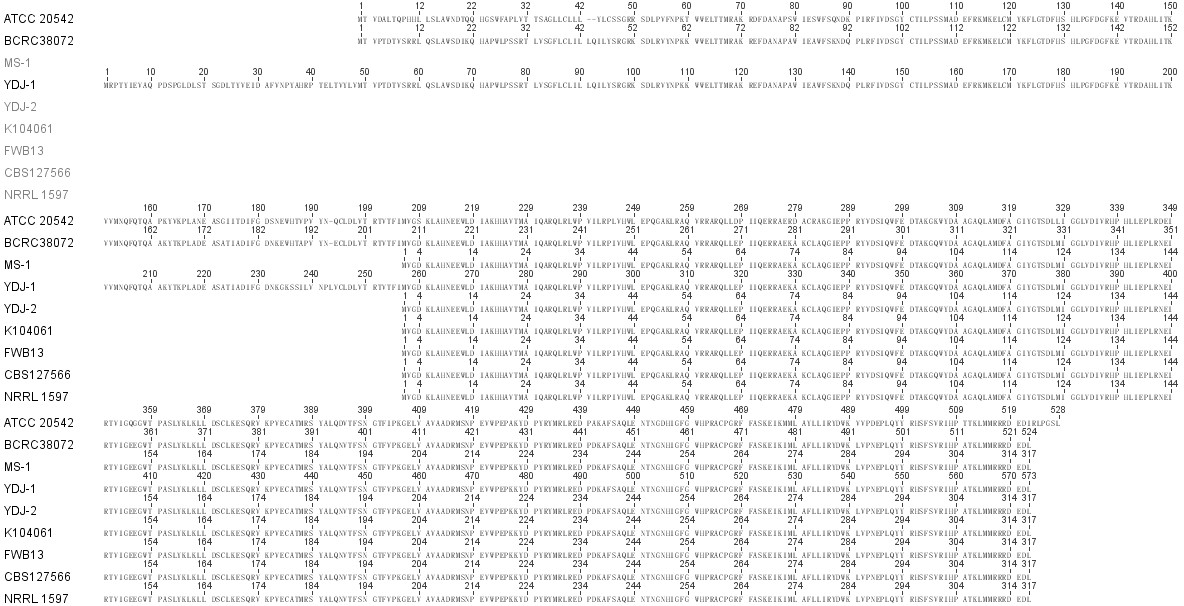

Supplement: Supplementary file 1 [file microorganisms-09-00747-s001.zip › microorganisms-1152183-supplementary-published (final)/Figure S4.jpg]

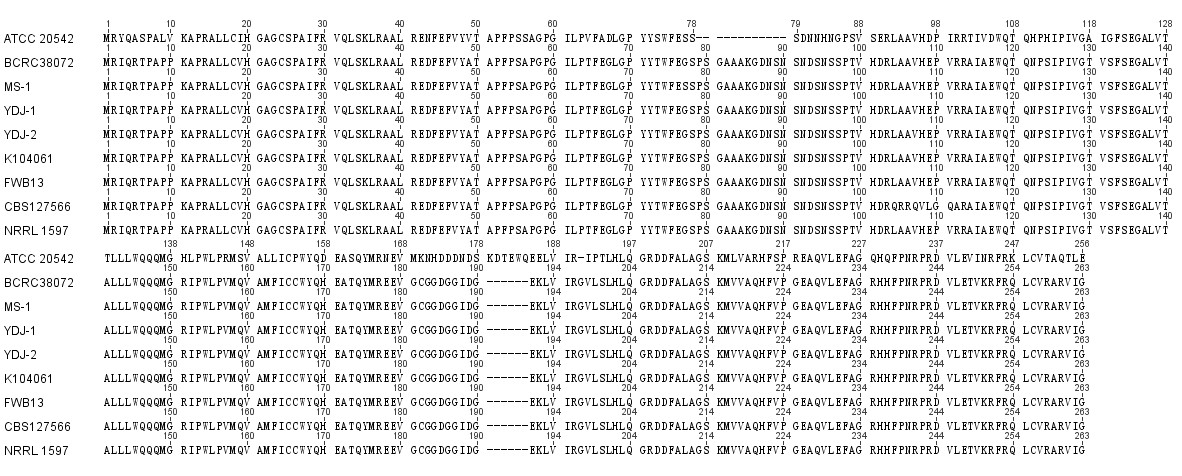

Supplement: Supplementary file 1 [file microorganisms-09-00747-s001.zip › microorganisms-1152183-supplementary-published (final)/Figure S5.jpg]

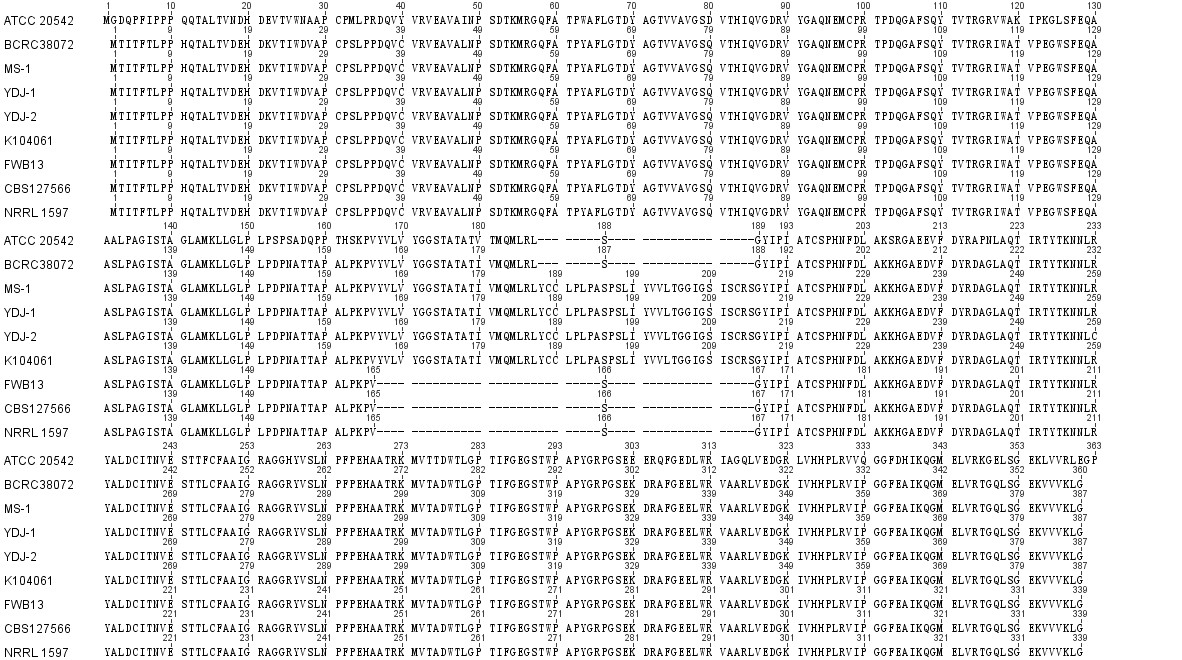

Supplement: Supplementary file 1 [file microorganisms-09-00747-s001.zip › microorganisms-1152183-supplementary-published (final)/Figure S6.jpg]

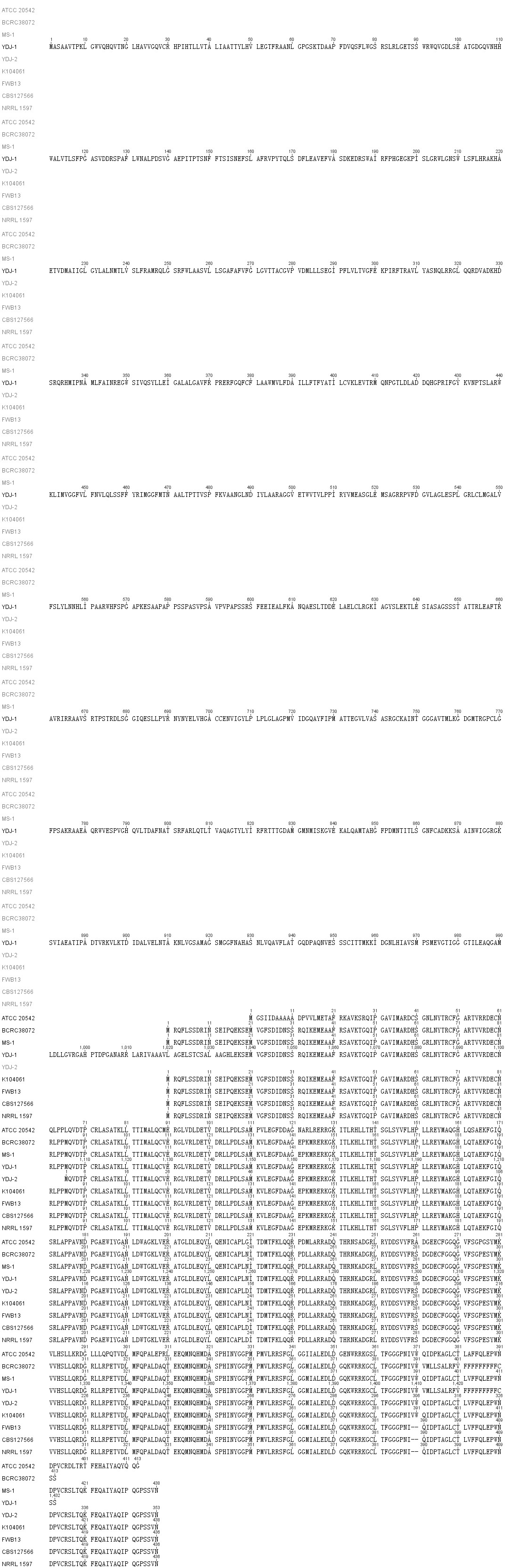

Supplement: Supplementary file 1 [file microorganisms-09-00747-s001.zip › microorganisms-1152183-supplementary-published (final)/Figure S7.jpg]

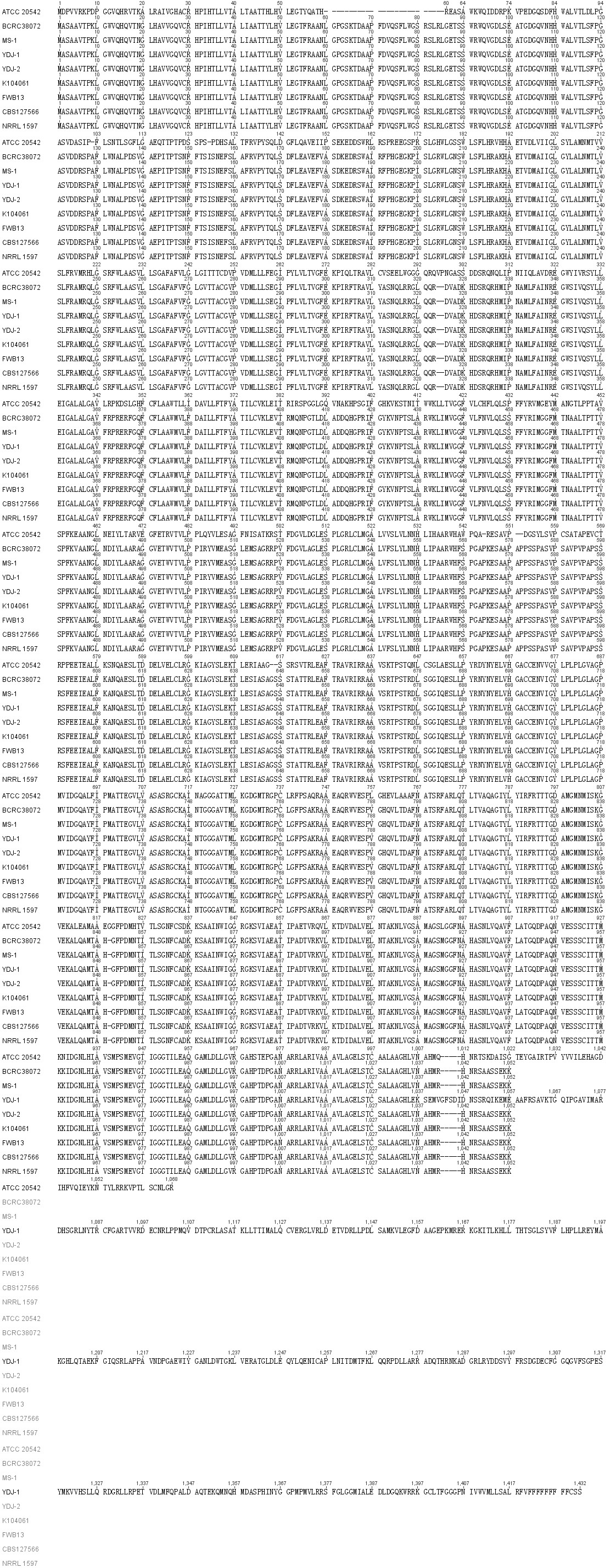

Supplement: Supplementary file 1 [file microorganisms-09-00747-s001.zip › microorganisms-1152183-supplementary-published (final)/Figure S8.jpg]

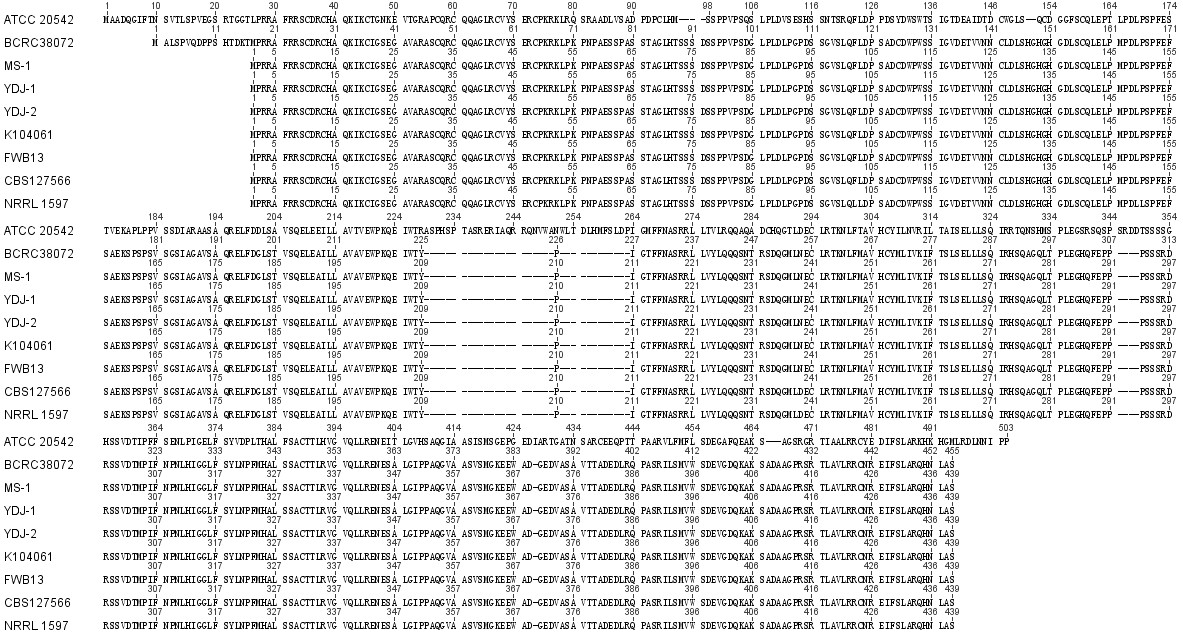

Supplement: Supplementary file 1 [file microorganisms-09-00747-s001.zip › microorganisms-1152183-supplementary-published (final)/Figure S9.jpg]
